# An Examination of the Relationship between Hotspots and Recombination Associated with Chromosome 21 Nondisjunction

**DOI:** 10.1371/journal.pone.0099560

**Published:** 2014-06-13

**Authors:** Tiffany Renee Oliver, Candace D. Middlebrooks, Stuart W. Tinker, Emily Graves Allen, Lora J. H. Bean, Ferdouse Begum, Eleanor Feingold, Reshmi Chowdhury, Vivian Cheung, Stephanie L. Sherman

**Affiliations:** 1 Department of Human Genetics, Emory University School of Medicine, Atlanta, Georgia, United States of America; 2 Department of Biology, Spelman College, Atlanta, Georgia, United States of America; 3 Department of Biostatistics, Graduate School of Public Health, University of Pittsburgh, Pittsburgh, Pennsylvania, United States of America; 4 Department of Human Genetics, Graduate School of Public Health University of Pittsburgh, Pittsburgh, Pennsylvania, United States of America; 5 Howard Hughes Medical Institute, University of Michigan, Ann Arbor, Michigan, United States of America; 6 Department of Human Genetics, University of Michigan, Ann Arbor, Michigan, United States of America; Duke University, United States of America

## Abstract

Trisomy 21, resulting in Down Syndrome (DS), is the most common autosomal trisomy among live-born infants and is caused mainly by nondisjunction of chromosome 21 within oocytes. Risk factors for nondisjunction depend on the parental origin and type of meiotic error. For errors in the oocyte, increased maternal age and altered patterns of recombination are highly associated with nondisjunction. Studies of normal meiotic events in humans have shown that recombination clusters in regions referred to as hotspots. In addition, GC content, CpG fraction, Poly(A)/Poly(T) fraction and gene density have been found to be significant predictors of the placement of sex-averaged recombination in the human genome. These observations led us to ask whether the altered patterns of recombination associated with maternal nondisjunction of chromosome 21 could be explained by differences in the relationship between recombination placement and recombination-related genomic features (i.e., GC content, CpG fraction, Poly(A)/Poly(T) fraction or gene density) on 21q or differential hot-spot usage along the nondisjoined chromosome 21. We found several significant associations between our genomic features of interest and recombination, interestingly, these results were not consistent among recombination types (single and double proximal or distal events). We also found statistically significant relationships between the frequency of hotspots and the distribution of recombination along nondisjoined chromosomes. Collectively, these findings suggest that factors that affect the accessibility of a specific chromosome region to recombination may be altered in at least a proportion of oocytes with MI and MII errors.

## Introduction

Trisomy 21, leading to Down Syndrome (DS), is the most common autosomal trisomy among live-born infants, occurring in approximately 1 in 700 live-births, and is caused mainly by the failure of chromosome 21 to properly segregate during oogenesis [1]. Increased maternal age and altered number and location of recombination events have been found to be associated with maternal meiotic errors involving chromosome 21 [2], [3]. Specifically, the absence of recombination [4] or the presence of a single recombinant event near the telomere of 21q [2] are associated with maternal meiosis I (MI) errors and these associations appear to be independent of the age of the oocyte (i.e., maternal age at the time of birth of the infant with trisomy 21) [5]. Meiosis II (MII) errors appear to be driven by different age and recombination traits: MII errors are associated with the placement of a recombinant event near the centromere of 21q [2] and this association increases with increasing age of the oocyte [5].

Studies of normal meiotic events in humans show that the placement of recombination is not a random event. Rather, both cis and trans-acting factors have been found to be associated with the placement of recombination. Specifically, GC content, CpG fraction and Poly(A)/Poly(T) fraction have each been found to be significant predictors of placement of sex-averaged recombination events in the human genome [6]. In addition, sequence variation in the zinc-finger domain of the gene *Proline Rich Domain Containing 9* (PRDM9) has a major impact on the location of recombination in humans [7], [8], [9], [10]. Specifically, allelic differences in the zinc finger binding domain of *PRDM9* explain approximately 80% of the heritable variation in “hotspot usage” ” (i.e. the frequency in which recombination occurs within linkage disequilibrium (LD) or “historically”-defined hotspots) [8], [11], [12]. The observation that both cis and trans-acting factors are associated with the placement of recombination led us to question whether the altered patterns of recombination associated with nondisjunction of chromosome 21 could be explained by differences in the relationship between recombination and genomic features (i.e., GC content, CpG fraction, Poly(A)/Poly(T) fraction or gene density) on 21q or differential hot-spot usage. This paper presents the first analyses of the relationship between recombination rate and the quantity of genomic features or LD-defined hotspots specifically along chromosome 21 in oocytes with a normal meiotic outcome, a MI nondisjunction error or a MII nondisjunction error.

## Materials and Methods

### Ethics Statement

The work presented in this publication was approved by the Emory Univeristy Institutional Review Board. All participants in provided written consent which indicated that the individual (1) agreed for study personnel to proceed with the interview and (2) consented for biological specimens to be obtained from them and their child. All information obtained during participant interviews and related to sample collection were catalogued electronically and de-identified.

### Trisomic Population

Families with an infant with full trisomy 21 were recruited through a multisite study of risk factors associated with chromosome mal-segregation [2], [13], [14]. Parents and the infant donated a biological sample (either blood or buccal) from which DNA was extracted. Only families in which DNA was available from both biological parents and the child with trisomy 21 were included, leading to a total of 297 maternal MI and 277 maternal MII cases of trisomy 21 (Table 1).

**Table 1:** Population Sample Sizes.

Meiotic Outcome Group and Recombination Type	Number of samples
MI Single	222
MI Proximal	75
MI Distal	75
MII Single	202
MII Proximal	75
MII Distal	75
Normal Single	1272
Normal Proximal	342
Normal Distal	342

### Trisomic Population Genotyping and Quality Control

Samples were genotyped at 1536 SNP loci on 21q by the Center for Inherited Disease Research using the Illumina Golden Gate Assay. The most centromeric SNP was rs2259403 (13,615,252 bp) and the most telomeric was rs7116 (46,909,248 bp). The average number of SNPs per 500 kb bin was 25.56 with a standard deviation of 25.91 with over 70% of cases exhibiting a recombinant having recombination breakpoints smaller than 1 Mb. Mendelian inconsistencies and sample mix-ups were identified using RelCheck among the trios. In addition, parental genotyping data were used to identify poorly performing SNPs. SNPs that met the following criteria were excluded from our analyses: minor allele frequency (MAF) <0.01, deviation from Hardy Weinberg Equilibrium (HWE) (p<0.01), heterozygosity >0.60 or > 10% missingness. We also excluded SNPs on a family-by-family basis if >50% of the genotype data for a proband had low intensity levels. As it relates to our exclusion of SNPs with a heterozygosity rate of >0.60, while we understand that is a very conservative/stringent cutoff, we did indeed examine the distribution of cases by stage and origin upon changing the heterozygosity rate and we did not see any significant changes in stage (data not shown). In addition, for a significant majority of our cases, stage and origin had been previously determined using STR data and compared to what was identified with our SNP only data.

### Determining Stage and Origin of Meiotic Chromosome Mal-Segregation

Individuals with trisomy 21 have three copies of chromosome 21 and thus display three alleles for each SNP genotyped on chromosome 21. In instances where trisomy 21 is caused by a maternal meiotic error, for each SNP examined, one of these alleles is inherited from dad, while the other two are inherited from mom. Maternal meiotic errors were confirmed upon determining that trisomic offspring inherited two alleles from mom and one from dad for SNPs genotyped on chromosome 21. Only cases of maternal origin were included in our analyses. Once the maternal origin of the meiotic error was established, markers located in the pericentromeric region (13,615,252 bp – 16,784,299 bp) of 21q were used to infer the stage of the meiotic error, MI or MII. If maternal heterozygosity was retained in the trisomic offspring, we concluded a MI error. If maternal heterozygosity was reduced to homozygosity, we concluded a MII error. In this assay, we cannot distinguish between the different types of underlying errors that might lead to an MII error. For example, sister chromatids that fail to separate during anaphase of MII or an error that is initiated in MI and not resolved properly in MII both lead to the contribution of sister chromatids to the gamete. Also, if sister chromatids prematurely separate in MI, some configurations will lead to both sister chromatids segregating to the same pole in MII. Lastly, when all informative markers in the parent of origin were reduced to homozygosity, the origin of nondisjunction was inferred to be a post-zygotic, mitotic error and excluded from the study.

### Identifying the Location of Recombination – Trisomic Samples

After genotyping quality control measures were implemented and SNP data were combined with STR data from our previous studies [3], we defined the location of recombinant events. The breakpoints of a single recombinant event were defined by a minimum of either one STR or eight consecutive, informative SNPs flanking the recombination breakpoint. An exception to this rule occurred when the most proximal or most distal informative markers on 21q indicated the presence of recombinant event. In these instances, a minimum of either one STR or four consecutive, informative SNPs were required to define the breakpoints of recombination. The presence of a double recombinant event was defined by a minimum of either one STR or 8 consecutive, informative SNPs flanking the recombination breakpoint on each side for both events.

### Euploid Population

SNP genotyping data for normally segregating chromosomes 21 were taken from families recruited for 1) the Autism Genetic Research Exchange (AGRE) (N = 743) [15], 2) the Framingham Heart Study (FHS) (N = 764) [16] and 3) the GENEVA Dental Caries Study (N = 107) [17] (Table 1). All families were two-generation families with a minimum of three children. This was necessary to define specific recombination profiles for each parent child transmission.

### Euploid Population Genotyping and Quality Control

The AGRE samples were genotyped for SNPs genome-wide using the Infinium(R) HumanHap550-Duo BeadChip. The AGRE data included genotypes at 520,017 markers genome-wide, however 11,473 markers were excluded from the analysis due to deviation HWE (p<10^−7^). After quality control measures were completed, there was genotype information for 7,810 SNPs on 21q for the AGRE dataset. The FHS samples were genotyped for SNPs genome-wide using the Genome-Wide Human SNP Array 5.0. The FHS data included genotypes at 500,568 markers. However, 22,000 markers were excluded from the analysis due to deviation from HWE (p<10^−7^). After quality control measures were completed, there was genotype information for 6,705 SNPs on 21q for the FHS dataset. The GENEVA samples were genotyped using the Illumina 610-Quad Array. The GENEVA dataset included genotypes at 620,901 SNPs. 58,610 markers were excluded from the analysis due to deviation from HWE (p<10^−5^), a MAF < 0.02. After quality control measures were completed, there was genotype information for 8,189 SNPs on 21q from the GENEVA population. All SNP locations were based on human NCBI Build 36 (hg18).

### Identifying the Location of Recombination – Euploid Samples

For the AGRE, FHS and GENEVA datasets, genotype data from members of two-generation families with three or more children were used to infer the location of recombination along the maternal chromosome 21. Our approach and software are described in Chowdhury et al. [18]. Briefly, parental genotypes were used to identify informative markers. Then, using these markers, genotypes of the children were compared to identify alleles inherited identical-by-descent from the mothers and fathers. Between two sibs, a switch from sharing the same maternal allele to not sharing was scored as a maternal recombination event.

### Examining the Relationship Between Genomic Features and Recombination

**Figure 1:**
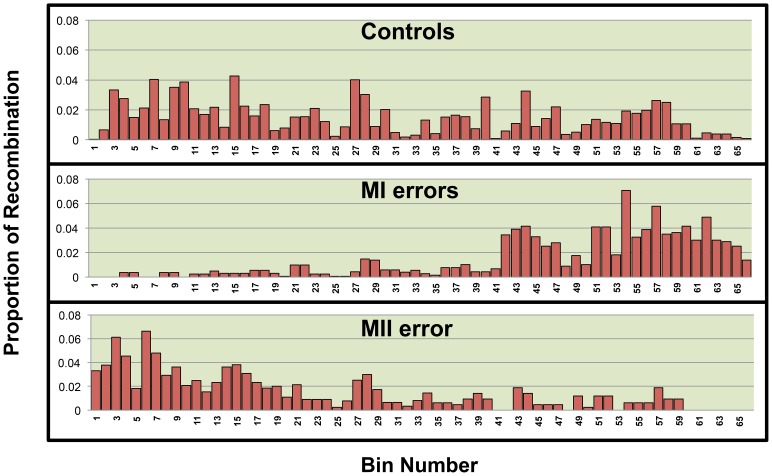
The distribution of single recombination events across the long arm of 21q by population. 21q was divided into 66 500

**Figure 2:**
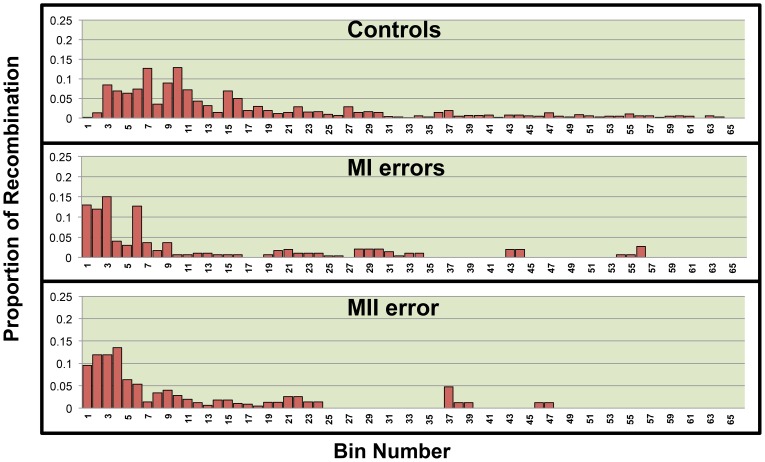
The distribution of the proximal recombinant of a double recombinant event across the long arm of 21q by population. 21q was divided into 66 500

**Figure 3:**
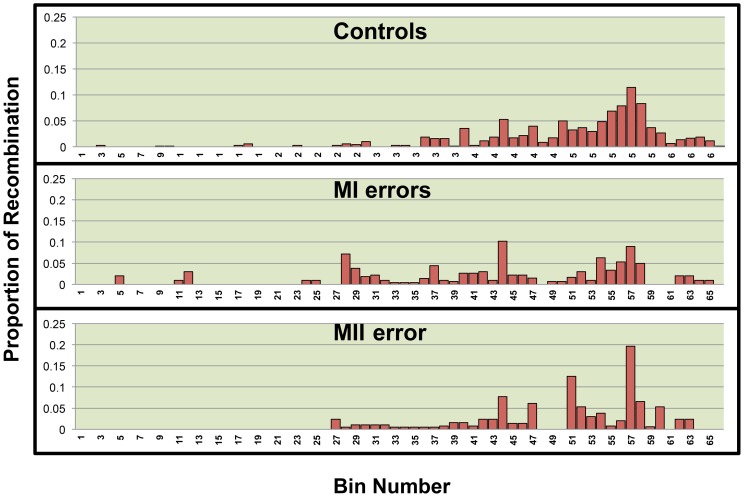
The distribution of the distal recombinant of a double recombinant event across the long arm of 21q by population. 21q was divided into 66 500

We used linear regression models to assess the relationship between the quantity of recombination and the quantity of each variable of interest found within regions across 21q. We divided 21q into 500 kb bins and calculated the quantity of each variable within a bin. We chose this bin size based on our level of refinement of recombination break-points. For the genomic features, we quantified the amount of each bin occupied by each genomic feature of interest, GC content, CpG content, Poly(A)/Poly(T) content were calculated as the proportion of each bin occupied by each feature however the number of genes per bin was calculated for gene density. Data on genomic features were based on the hg18 build of the human genome and retrieved from the following tables within the USCS Genome Browser: gc5Base, CpGIslandExt and rmsk (repeat master), UniGene_3 and RefGene. As for hotspots, we used the number of LD-defined hotspots, as defined by Myers et. al.[19] per bin as the predictor variable (Figure S1). The outcome variable was defined as the proportion of all chromosome 21 single or double recombinant events that occurred within the bin. As it is well known that single recombination events cluster in the telomeric and centromeric regions of 21q for the MI and MII error groups (Fig. 1), respectively, we included bin location as a variable in our models as it may be a confounding variable. We stratified analyses by chromosomes with single and double recombinant events (Figs. 2 and 3, Table 1) as mechanisms of chromosome 21 nondisjunction may differ based on the number of recombinant events on 21q[2], [3], [5], [14]. Univariate linear regression was then used to determine whether there was significant correlation between the quantity of each predictor variable and the proportion of recombination within a bin (p≤0.05).

General linear regression models were used to test for differences in the slopes of the regression models between comparison groups (MI or MII versus Controls) for each predictor. That is, to compare MI error to normal meiotic outcomes, we included the interaction term of comparison group by genomic feature within a bin. This type of model was also used to compare MII errors with controls. Once again, we included bin location as a covariate for the reason stated above.

### Data Availability

Data on recombination along normally segregating chromosomes 21 came from three different studies, the AGRE, FHS and GENEVA. Access to data used in this analysis from the AGRE is publically available upon IRB approval or exemption. For more information please logon to https://research.agre.org. Data from the FHS and GENEVA Studies is now available via dbGaP, accession numbers phs000007.v23.p8 and phs000440.v1.p1 respectively. Genotypes used to determine the placement of recombination along nondisjoined chromosomes 21 will also be available via dbGAP.

## Results

### Association between genomic features along chromosome 21q and the proportion of recombination events

We first examined meiotic events with one detectable recombinant event on 21q (Table 2). In regression models that included both the specific genomic feature (i.e., GC content, CpG fraction, Poly(A)/Poly(T) fraction or gene density) and the location of the bin along 21q, only location, was found to be a significant predictor of the amount of recombination for the vast majority of features. This is consistent with previous work that has established altered placement of recombination as a significant risk factor for chromosome 21 nondisjunction [2], [3]. There was one exception to this pattern: among the MII errors with a single recombinant, both location and GC content were significant predictors of the amount of recombination. This suggests that among MII single recombinant events, where the increased risk is associated with a pericentromeric recombinant, there may be a preference for recombination to occur in regions with elevated GC content and close to the centromere.

**Table 2:** Values of slopes/beta coefficients for GC, CpG, PolyAT and gene denisty for single recombinants stratified by meiotic outcome group.

Predictor Variable	Controls	MI	MII
GC	−0.0108	0.0377	0.0856*
21q location	−0.0002	0.0006*	−0.0008*
CpG	−0.145	−0.3102	0.161
21q location	−0.0002	0.0008*	−0.0006*
Poly(A)/Poly(T)	−0.0354	−0.1311	−0.4959
21q location	−0.0002*	0.0006*	−0.0007*
Gene Density	−0.0005	−0.0041	0.0044
21q location	−0.0002*	0.0007*	−0.0006*

Beta values for each genomic feature adjusted for bin variable. Beta coefficients/slopes that are significantly different from zero are marked with an asterisk (p<0.05)*.

We then looked at meiotic events with two detectable recombinants and separated the analyses by the proximal and distal event. For proximal recombinant events (Table 3), GC and CpG content as well as bin location were found to be positively correlated with recombination among MI and MII errors; no association for these features was found among normal meiotic control recombinant events of this type (Table 3). Poly(A)/Poly(T) fraction was found to be inversely correlated with the amount of recombination among MI and MII errors and normal outcomes. Collectively these observations suggest that MI and MII proximal recombinant events occur in GC rich regions more often than statistically expected if there was no relationship between the amount of recombination and GC (or CpG) content. We did not find any associations between genomic features and recombination among our MI and MII distal recombination events (Table 4).

**Table 3:** Values of slopes/beta coefficients for GC, CpG, PolyAT and gene density for the proximal recombinant of a double recombinant event stratified by meiotic outcome group.

Predictor Variable	Controls	MI	MII
GC	0.167	0.366*	0.477*
21q location	−0.001*	−0.002*	−0.002*
CpG	0.36	0.891*	1.099*
21q location	−0.001	−0.001*	−0.001*
Poly(A)/Poly(T)	−1.739*	−3.237*	−3.612*
21q location	−0.001*	−0.001*	−0.002*
adjusted Gene Density	0.0137	0.019	0.005
21q location	−0.001*	−0.001*	−0.001*

Beta values for each genomic feature adjusted by bin variable. Beta coefficients/slopes that are significantly different from zero are marked with an asterisk (p<0.05)*.

**Table 4:** Values of slopes/beta coefficients for GC, CpG, Poly(A)/Poly(T) and gene density for the distal recombinant of a double recombinant event stratified by meiotic outcome group.

Predictor Variable	Controls	MI	MII
GC	−0.039	−0.098	0.037
21q location	0.001*	0.001*	0.001*
CpG	−0.705*	−0.573	−0.558
21q location	0.001*	0.001*	0.001*
Poly(A)/Poly(T)	−0.215	0.195	−0.794
21q location	0.001*	0.0004*	0.001*
Gene Density	0.002	0.001	−0.008
21q location	0.001*	0.0004*	0.001*

Beta values for each genomic feature adjusted by bin variable. Beta coefficients/slopes that are significantly different from zero are marked with an asterisk*.

### Hotspot usage among normally disjoined chromosome 21 events

We examined LD-defined hotspots first among normally disjoining chromosomes (controls). We looked separately at those with one recombinant event and those with two recombinant events. Among those with one detectable event, we found a significant positive association between the number of hotspots per bin and the proportion of recombination per bin (p<.0001) (Table 5). Similarly, among those with two detectable events, we found that the proportion of proximal and distal recombinant events within a bin was significantly associated with LD-defined hotspots density (p = 0.001 and <.0001, respectively, Table 5). Thus, as expected, the amount of recombination per bin is positively correlated with historical hotspot density suggesting that historical hotspots are used for recombination along normally segregating chromosomes 21.

**Table 5:** Beta coefficient/slope values for hotspots variable adjusted by bin variable and stratified by meiotic outcome group and number of recombinants on chr21.

Recombination Type	Predictor Variable	Controls	MI	MII
Single Recombination	Hotspot count	0.002*	0.002*	0.001*
	Bin location	−0.0002*	0.0007*	−0.0006*
Double Recombination -Proximal	Hotspot count	0.003*	−0.0005	0.0002
	Bin location	−0.001*	−0.0009	−0.001
Double Recombination - Distal	Hotspot count	0.0034*	0.002*	0.0028*
	Bin location	0.0007*	0.0004*	0.0007*

Beta coefficients/slopes that are significantly different from zero are marked with an asterisk*.

### Hotspot usage among nondisjoined chromosome 21events due to MI errors

**Figure 4:**
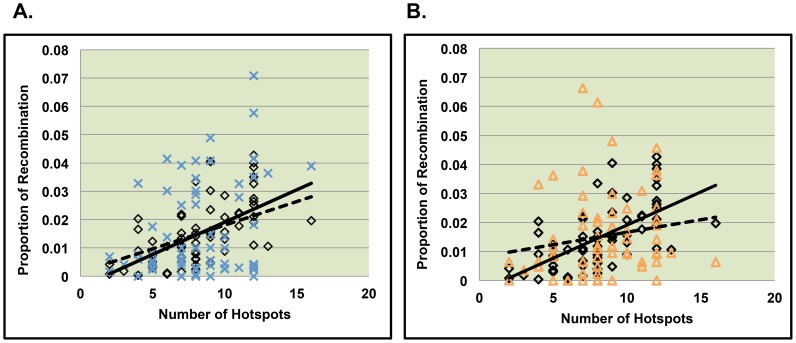
Comparison of the relationship between hotspot usage between MI and MII cases and Controls. Figure 4A and 4B represent MI and MII cases respectively with only one recombinant event on 21q. The solid line represents the relationship between the number of hotspots per bin and the proportion of recombination per bin along normally segregating chromosomes 21. The dotted line represents the relationship between the number of hotspots per bin and the proportion of recombination per bin along chromosomes 21 from MI errors (figure 4A) and MII errors (figure 4B).

We first examined single recombinants along 21q. Similar to normally segregating chromosomes, we found hotspot density to be a positively correlated with the proportion of recombination within a bin (p = 0.0006, Table 5). In order to determine whether the strength of the relationship between recombination and hotspot density differed between control and MI single recombinant events, we next tested whether the strength of the association between the proportion of recombination and hotspot density among MI errors significantly differed from that of controls and found no evidence for different patterns (Fig. 4A, p = 0.43).

**Figure 5:**
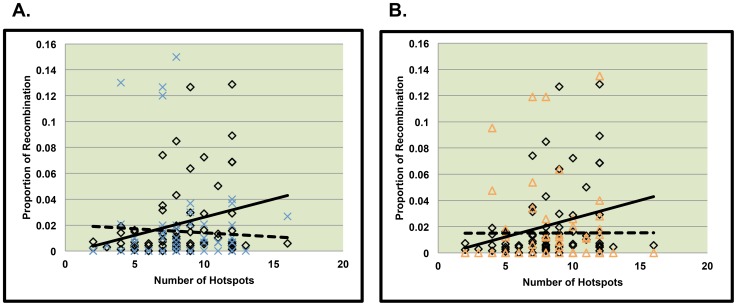
Comparison of slopes between MI or MII errors and controls for the proximal recombinant of double recombinant events. Figures 5A and 5B represent data from the proximal recombinant event of chromosomes displaying two recombinant events on 21q. The solid line represents the relationship between the number of hotspots per bin and the proportion of recombination per bin along normally segregating chromosomes 21. The dotted line represents the relationship between the number of hotspots per bin and the proportion of recombination per bin along chromosomes 21 from MI errors (figure 5A) and MII errors (figure 5B).

**Figure 6:**
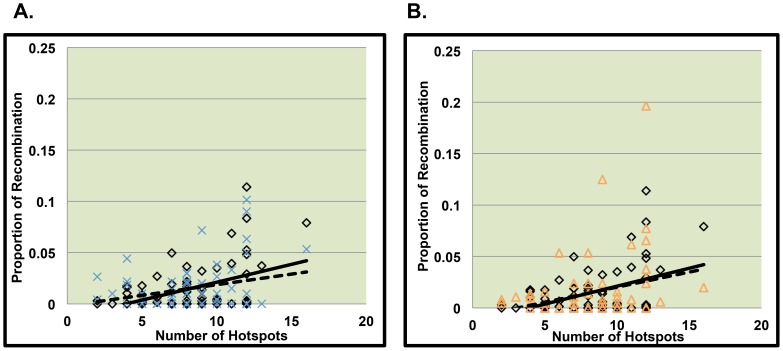
Comparison of slopes between MI or MII errors and controls for the distal recombinant of double recombinant events. Figures 6A and 6B represent data from the distal recombinant event of chromosomes displaying two recombinant events on 21q. The solid line represents the relationship between the number of hotspots per bin and the proportion of recombination per bin along normally segregating chromosomes 21. The dotted line represents the relationship between the number of hotspots per bin and the proportion of recombination per bin along chromosomes 21 from MI errors (figure 6A) and MII errors (figure 6B).

Among nondisjoining chromosomes with two detectable recombinants we separated analyses by the proximal and distal event. We did not detect a significant relationship between hotspot density and the proportion of recombination per bin for proximal recombinants (fig. 5A, table 5). For distal recombinant events, we found that recombination was significantly associated with LD-defined hotspot density (Table 5, p = 0.02), however for the patterns of association did not differ between MI and controls (Fig. 6A, p = 0.21).

### Hotspot usage among nondisjoined chromosome 21events due to MII errors

As for MII, we detected a significant positive correlation between hotspot density and the proportion of recombination across 21q for single recombinants. The association patterns differed significantly from that of controls (Fig. 4B, p = 0.01), with MII single recombinant events being less correlated with hotspot density than controls. Among MII errors with two recombinant events, as with MI errors, we did not detect a significant correlation between the proportion of recombination per bin and the density of LD-defined hotspots in the proximal region (fig 5B, table 5). For MII distal events, there was a significant positive association between LD-defined hotspot density and the proportion of recombination per bin (Table 5, p = 0.02). The association patterns did not differ significantly between MII versus control events (Fig. 6B, p = 0.69).

## Discussion

### Association between genomic features along chromosome 21 and the proportion of recombination events along 21q

In our analysis of the relationships between our genomic features of interest and the proportion of recombination per bin, we found several genomic features to be associated with recombination, although these results were not consistent among recombination types (single, double proximal or distal event). Based on the lack of patterns, we were unable to draw any significant conclusions. We do note that our large sample of normal maternal meiotic events (n = 1,272) for 21q did not show many of the relationships found in the study of Kong et al.[20]. We attribute this to a difference in the study design, not to the sample size, as both studies had comparable numbers of meiotic events. First, we restricted our analysis to 21q, whereas the original associations were found through the analysis of the entire genome. Second, the Kong et al. study the sex-averaged associations based on 628 paternal and 629 maternal meiotic outcomes; we only examined maternal recombination events. Taken together, a study of sex-specific, chromosome-specific associations of genomic features and recombination may provide further insights into the control of recombination.

### Hotspot usage among nondisjoined chromosome 21 events

Our findings with regard to LD-defined historical hotspots differ between our meiotic outcomes groups and provide some insight into recombination-associated nondisjunction. First, we gain confidence that our analyses are able to identify associations with hotspot usage, as our findings from normally disjoining chromosomes 21 are consistent with expectation. That is, using our sample of normal meiotic events, our statistical analysis showed the expected pattern of increased recombination in the LD-defined hotspots for single events and double recombinant events on 21q. As it relates to MI errors, our analysis of single recombinants indicated an association of recombination with the distribution of LD-defined hotspots along 21q, similar to controls, suggesting that these events occur preferentially near or within LD-defined hotspots. This is interesting as our previous studies have shown that the average location of MI single recombinant events is approximately 10 Mb closer to the telomere of 21q than normal single recombinant events [21]. As a result, it does not appear that the altered patterns of recombination associated with MI errors can be explained by differential hotspot usage.

We found different patterns of association for MII single recombinant events compared with those for MI-single recombinants events and controls. Specifically, we found that the proportion of single recombinants across 21q per bin is significantly correlated with LD-defined hotspots; however, this association is not as strong as it is in controls. From our most recent work, recombination along 21q among MII errors is more proximally located: average location 22.60 Mb on 21q compared with 27.53 Mb on 21q among normal events [21]. Potentially factors characteristic of pericentromeric DNA such as chromatin structure or epigenetic modifications may affect the accessibility of a specific chromosome region to recombination in at least a proportion of oocytes with meiotic errors.

In our analysis of double recombinants events, we found similar results with respect LD-defined hotspots among MI and MII errors. We detected a significant relationship between LD-defined hotspots for the distal recombinant events among doubles, but not the proximal events. Furthermore, the lack of evidence for an association in the proximal region differed from that in controls where an association was detected (i.e., significant interaction). Oliver et al. [21]. found that the unusual pericentromeric proximal events imposed a risk for MII nondisjunction and were associated with increased maternal age, but this pattern was not found among MI errors. Further work is needed to synthesize these results with those based on location of the events along nondisjoined chromosomes 21.

We do not provide consistent evidence that genomic features present at the site of recombination or differential hotspot usage are implicated in the nondisjunction of chromosome 21. However, altered patterns of recombination on 21q have long-been identified to be associated with an increased risk for chromosome 21 nondisjunction. Thus we believe that either the absence or altered physical placement of recombination may be more important as it relates to the risk for chromosome 21 nondisjunction. Moving forward we plan to take a genome-wide approach in efforts to identify genetic factors implicated in the altered patterns of recombination associated with chromosome 21 nondisjunction.

## Supporting Information

Figure S1 Distribution of Hotspots along 21q. Hotspot counts for each of the 66 bins across 21q.
(TIF)
