# Supplementary figures and images for: An Examination of the Relationship between Hotspots and Recombination Associated with Chromosome 21 Nondisjunction
Source: PLoS One. 2014 Jun 13;9(6):e99560. doi: 10.1371/journal.pone.0099560 (PMC4057233; doi:10.1371/journal.pone.0099560)

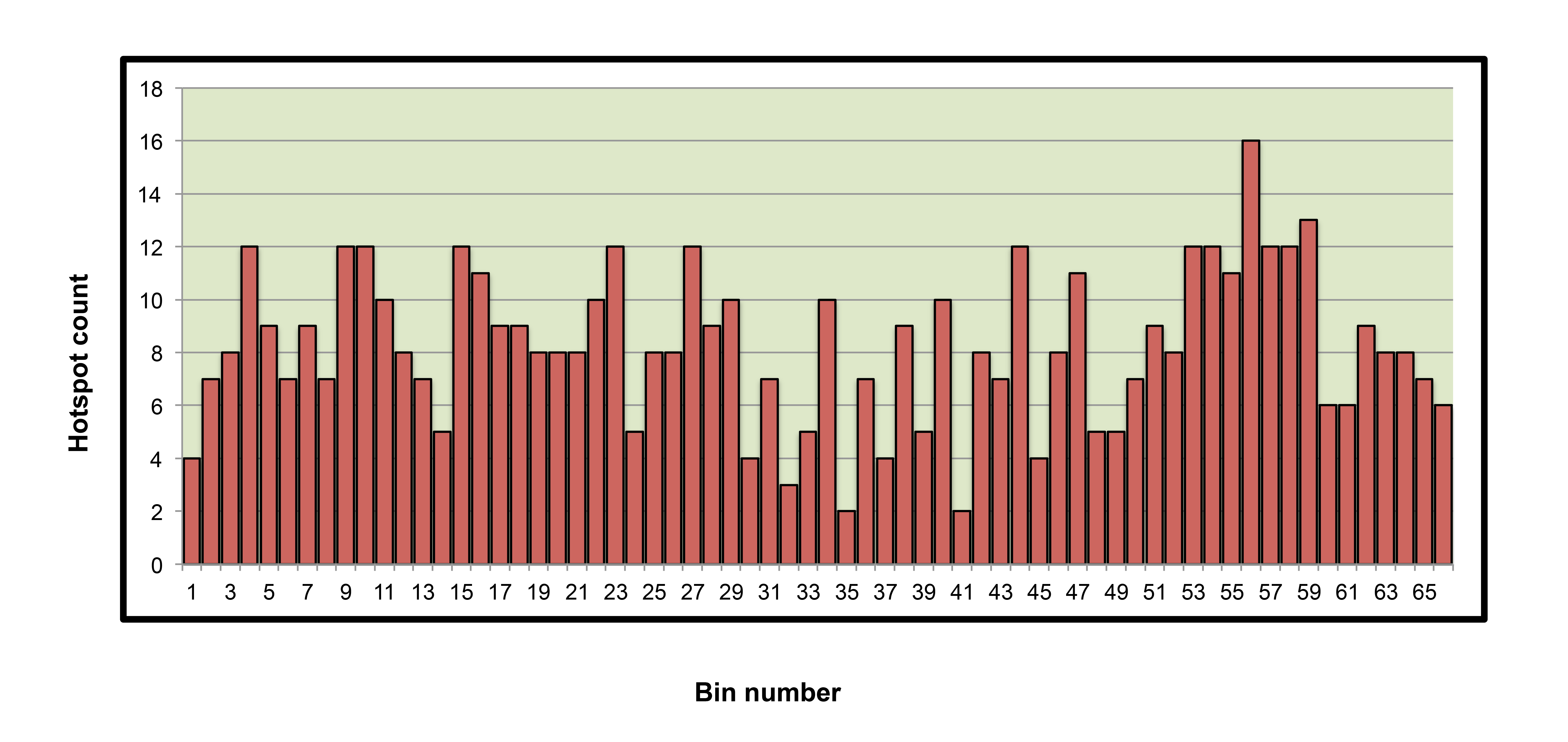

Supplement: Figure S1 — Distribution of Hotspots along 21q. Hotspot counts for each of the 66 bins across 21q. (TIF) [file pone.0099560.s001.tif]
